# Identification of necroptosis‐related gene signature and characterization of tumour microenvironment infiltration in non‐small‐cell lung cancer

**DOI:** 10.1111/jcmm.17494

**Published:** 2022-07-24

**Authors:** Juji Dai, Yangyang Fu

**Affiliations:** ^1^ Department of Colorectal and Anal Surgery the First Affiliated Hospital of Wenzhou Medical University Wenzhou China; ^2^ Division of Pulmonary Medicine The First Affiliated Hospital of Wenzhou Medical University, Key Laboratory of Heart and Lung Wenzhou China

**Keywords:** bioinformatics, necroptosis, non‐small‐cell lung cancer, prognostic signature, tumour immune microenvironment

## Abstract

Necroptosis is a programmed necrosis in a caspase‐independent fashion. The role of necroptosis‐related genes (NRGs) in lung cancer remains unknow. Herein, we classified TCGA‐LUAD cohort into two necroptosis‐related subtypes (C1 and C2) by consensus clustering analysis. The result showed that subtype C1 had a favourable prognosis and higher infiltration levels of immune cells. Moreover, subtype C1 was more activated in immune‐associated pathways. Then, we established an NRG prognosis model (NRG score) composed of six NRGs (RIPK3, MLKL, TLR2, TLR4, TNFRSF1A, NDRG2) and divided the cohort into low‐ and high‐risk group. We found that the NRG score was associated with prognosis, tumour immune microenvironment and tumour mutation burden. We also constructed an accurate nomogram model to improve the clinical applicability of NRG score. The result indicated that NRG score may be an independent prognostic marker for lung cancer patients. Taken together, we established a prognosis model that may deepen the understanding of NRGs in lung cancer and provide a basis for developing more effective immunotherapy strategies.

## 1 INTRODUCTION

Lung cancer is the leading cause of cancer death and a heavy burden in every country of the world.^1^, ^2^ Non‐small‐cell lung cancer (NSCLC) is the most common type, which accounts for nearly 80%. Although great advances have been made in the treatment of lung cancer, the 5‐year survival rate is only 10%–20%.^2^ Tumour microenvironment (TME) is the internal environment that supports the survival and development of tumour cells. It includes vasculature, cancer‐associated fibroblasts (CAFs), extracellular matrix (ECM) and infiltrating immune cells. Specific TME may potentially be involved in tumour stage, clinical outcome and therapeutic responses.^3^, ^4^, ^5^ Therefore, identification of novel biomarkers and molecular targets of TME cell infiltration may predict the response to immunotherapy and provide a comprehensive understanding of the underlying mechanism of lung tumorigenesis.

Necroptosis is a programmed necrosis mediated by receptor‐interacting protein kinase 1 (RIKP1), receptor‐interacting protein kinase 3 (RIKP3) and mixed lineage kinase domain‐like pseudokinase (MLKL).^6^ A diverse range of stimuli, including tumour necrosis factor receptor (TNFR), T‐cell receptors (TCRs) and various chemotherapy drugs, have been involved in the activation of necroptosis.^7^ Accumulating evidence have reported that necroptosis has a dual effect on cancer biology, especially in cancer immunity. On one hand, necroptosis is shown to capable of inducing immune response tolerance or pro‐tumorigenic inflammation.^8^, ^9^, ^10^ On the other hand, necroptosis helps to strengthen the immune ability of anticancer drugs.^11^ Decreased expression of necroptosis factors has been found in NSCLC tissues and was associated with worse prognosis in NSCLC patients.^12^, ^13^ The activation of necroptosis pathway has been shown to remarkably increased the killing ability of lung cancer cell mediated by chemotherapy drugs or radiation.^14^, ^15^, ^16^ Collectively, necroptosis may become a novel approach in cancer therapy.

In this study, we mined the Cancer Genome Atlas (TCGA) and Gene Expression Omnibus (GEO) databases to construct a necroptosis‐related genes (NRGs) score, which may play an important role in predicting the prognosis and the immune infiltration level of lung cancer. Meanwhile, we found the potential biological processes and signalling pathways that may be involved in necroptosis.

## 2 MATERIALS AND METHODS

### 2.1 Data sources and preprocessing

The process of this work was presented in Figure S1. Gene expression, clinicopathological characteristics and prognostic information were downloaded from TCGA database (https://portal.gdc.cancer.gov/). Five hundred lung adenoma tissues and fifty‐nine normal tissues were obtained for the following analyses. Detailed information of TCGA‐LUAD cohort is listed in Table S1. The validation cohort was acquired from GEO database (https://www.ncbi.nlm.nih.gov/geo/). Seventeen necroptosis‐related genes (RIPK1, RIPK3, MLKL, ALDH2, NDRG2, TLR2, TLR3, TLR4, TNFRSF1A, PGAM5, ZBP1, NR2C2, HMGB1, EZH2, CXCL1, USP22, TRAF2) were obtained from the published literature.^17^, ^18^, ^19^, ^20^, ^21^, ^22^, ^23^, ^24^, ^25^

### 2.2 Consensus clustering analysis

Cluster analysis was performed using ConsensusClusterPlus. *K* = 2 was determined as the optimal number of the cluster according to empirical cumulative distribution function plot. Then, principal component analysis (PCA) was performed using the ‘prcomp’ package in R software.

### 2.3 Correlation between clinicopathological characteristics and prognosis

The relationship between clinicopathological features and prognosis was assessed by Chi‐ squared test. The clinicopathological features included age, stage, recurrence and KRAS mutation. The survival curves of different subtypes were analysed using Kaplan–Meier curves.

### 2.4 Functional enrichment analysis

Gene set variation analysis (GSVA) was performed with the gene subset (c2.cp.kegg.v7.4.symbols.gmt) downloaded from the MsigDB database (http://www.gsea‐msigdb.org/gsea/downloads.jsp). The differentially expressed genes (DEGs) between the two necroptosis subtypes were identified using the ‘limma’ package in R software. The cut‐off criteria for DEGs were an absolute fold change more than1.5 and a *p*‐value <0.05. Then, Gene Oncology (GO) and Kyoto Encyclopedia of Genes and Genomes (KEGG) analysis were performed using the ‘clusterProfiler’ package in R software.

### 2.5 Assessment of the tumour immune microenvironment

ESTIMATE algorithm was performed to calculate the immune and stromal scores of each sample. CIBERSORT algorithm was also performed to evaluate the fractions of 25 tumour‐infiltrating immune cells.

### 2.6 Construction of the NRG prognostic signature

Univariate Cox analysis was applied to screen the necroptosis‐related prognostic genes. Then, Lasso Cox regression analysis was utilized to establish a necroptosis‐related prognostic gene model. At last, risk score was calculated with the following formula (sum of coefficients x necroptosis‐related gene expression), and patients were stratified into high‐ and low‐risk subtypes with a median threshold. The Kaplan–Meier analysis was used to analyse the prognosis of each patient, and receiver operating characteristic (ROC) analysis was performed to assess the predictive performance of this prognostic signature.

### 2.7 Construction of the nomogram model

The clinicopathological features and risk score were applied to establish a predictive nomogram model using the ‘rms’ package. Time‐dependent ROC curves for 1‐, 3‐ and 5‐year survivals were performed to evaluate the nomogram.

### 2.8 Statistical analyses

All statistical analyses were performed with R version 4.1.0. Pearson correlation tests were used to analyse the correlation of immune infiltration levels. Statistical significance was defined as *p* < 0.05.

## 3 RESULTS

### 3.1 Consensus clustering of necroptosis‐related patterns in TCGA‐LUAD cohort

**FIGURE 1:**
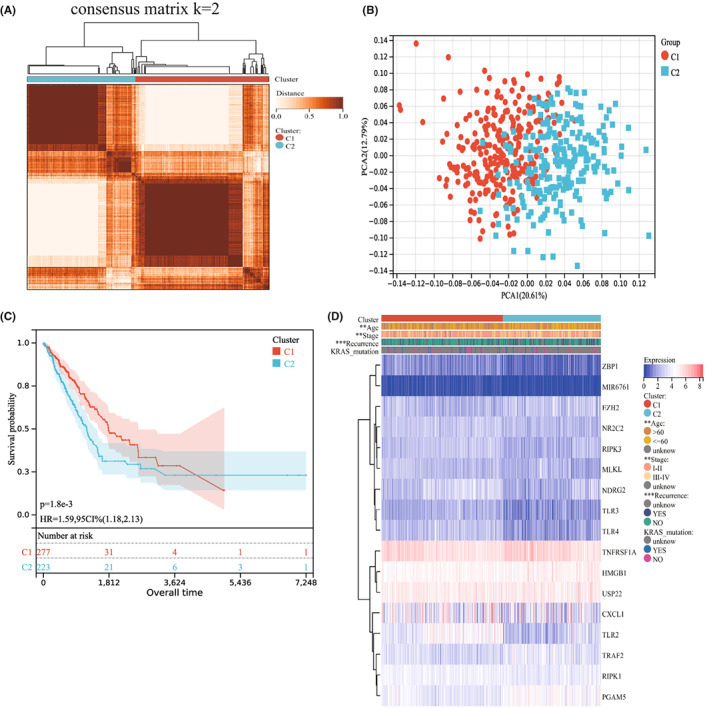
Consensus clustering and clinicopathological features of necroptosis‐related patterns in TCGA‐LUAD cohort. (A) Consensus matrix heatmap of TCGA‐LUADUnidentified cohort. (B) PCA analysis of C1 and C2 subtypes. Red dots represents patients in subtype C1, green dots represent patients in subtype C2. (C) Kaplan‐Meier curves of C1 and C2 subtypes. The optimal cut‐off value for the categories was median. Logrank *p* < 0.001. (D) Heatmap of clinicopathological characteristics and NRGs expression of C1 and C2 subtypes. *p < 0.05; **p < 0.01; ***p < 0.001

We performed consensus clustering on TCGA‐LUAD cohort based on seventeen NRGs expression. The result showed that *k* = 2 is the optimal number for diving the whole cohort into two subtypes, C1 (*n* = 277) and C2 (*n* = 223), according to the cumulative distribution function curve (Figure 1A). Meanwhile, PCA analysis result showed that there was an obvious difference in the transcriptional profiles between the two subtypes (Figure 1B). The Kaplan– Meier curves revealed that subtype C2 had a worse prognosis compared with subtype C1 (*p* < 0.001; Figure 1C). Further, we sought to the relationship between clinicopathological features and the two different subtypes. The results indicated that the subtype C2 was preferentially associated with higher age (*p* < 0.01), higher TNM stage (*p* < 0.01) and increased recurrence risk (*p* < 0.001) (Figure 1D).

### 3.2 Features of tumour immune microenvironment in different subtypes

**FIGURE 2:**
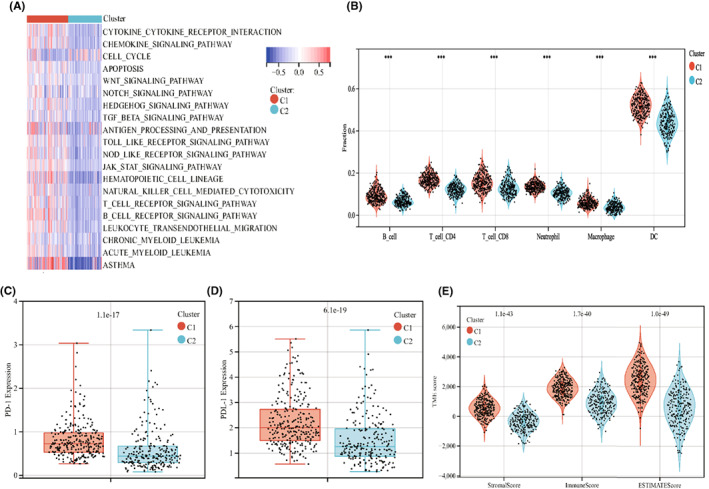
Comparison of tumor immune microenvironment between two subtypes. (A) GSVA analysis results of C1 and C2 subtypes. Red represents activated pathway, blue represents inhibited pathway. (B) Infiltration levels of tumor immune cells in C1 and C2 subtypes. ***p < 0.001. (C) Expression of PD‐1 in C1 and C2 subtypes. ***p < 0.001. (D) Expression of PDL‐1 in C1 and C2 subtypes. *p < 0.05; **p < 0.01; ***p < 0.001

GSVA enrichment analysis showed that the immune‐associated pathways significantly enriched in subtype C1, including cytokine receptor interaction, chemokine signalling pathway, TGF‐β signalling pathway, antigen processing, Toll‐like and NOD‐like receptor signalling pathways, JAK–STAT signalling pathway, natural killer cell‐mediated cytotoxicity, T‐ and B‐cell receptor signalling pathway (Figure 2A). Consistent with this result, the infiltration levels of immune cells were much higher in subtype C1, compared with the subtype C2 (Figure 2B). Moreover, the expression of programmed cell death protein 1(PD‐1) and programmed cell death protein ligand1 (PD‐L1), which are critical immune checkpoints in lung cancer, were increased in subtype C1 than those in subtype C2 (Figure 2C, D). We also investigated the TME score of the two subtypes using the ESTIMATE package. The result showed that the levels of stromal scores, immune scores and ESTIMATE score were obviously higher in subtype C1, compared with subtype C2 (Figure 2E).

### 3.3 Establishment and validation of the prognostic NRG score

**FIGURE 3:**
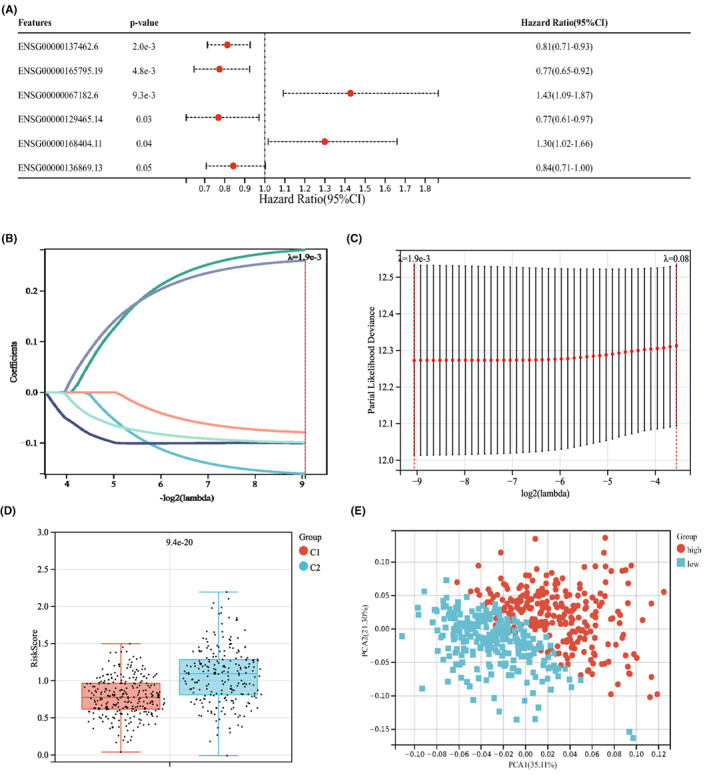
Identification of representative prognostic genes and PCA analysis. (A) Forest plot analysis of six prognostic genes. (B, C) Lasso Cox regression analysis and partial likelihood deviance on the prognostic genes. (D) Correlation between C1, C2 subtypes and risksores. ***p < 0.001. (E) PCA analysis of low‐risk and high‐risk groups

**FIGURE 4:**
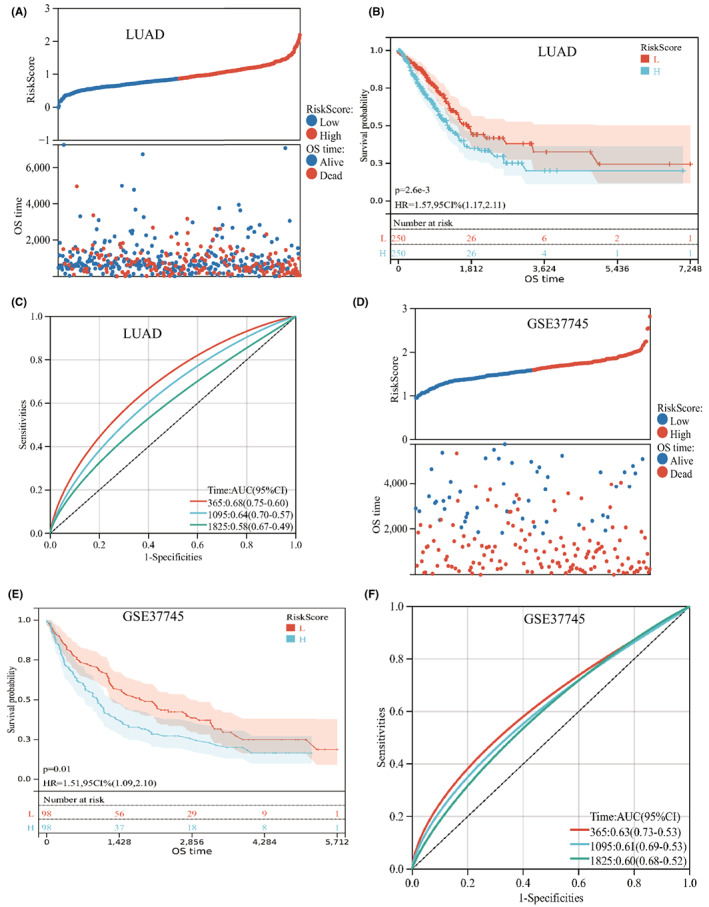
Construction and validation of NRG score in training and testing set. (A) Distribution risk score and survival status in TCGA‐LUAD cohort. (B) Kaplan‐Meier curves of low‐risk and high‐risk groups in TCGA‐LUAD cohort. Logrank *p* < 0.001. (C) ROC curves of NRG score in TCGA‐LUAD cohort. (D) Distribution of risk score and survival status in GSE37745 set. (E) Kaplan‐Meier curves of low‐risk and high‐risk groups in GSE37745 set. Logrank *p* < 0.05. (F) ROC curves of NRG score in GSE37745 set. *p < 0.05; **p < 0.01; ***p < 0.001

We constructed an NRG prognostic model that may be used to predict the prognosis of NSCLC patients effectively. Univariate COX regression analysis was performed with a total of seventeen NRGs mentioned above. Lasso COX regression analysis was subsequently used to decrease the overfitting risk. We eventually obtained a prognostic necroptosis‐associated risk signature consist of six NRGs (RIPK3, MLKL, TLR2, TLR4, TNFRSF1A, NDRG2) (Figure 3A–C). The NRG score was determined as follows: Risk score = (−0.1605 × expression of RIPK3) + (0.2812 × expression of MLKL) + (−0.0998 × expression of TLR2) + (−0.0788 × expression of TLR4) + (0.2601 × expression of TNFRSF1A) + (−0.0987 × expression of NDRG2). Then, every patient was assigned a risk score based on the abovementioned formula. We observed a significant difference in NRG score between the subtype C1 and subtype C2. Compared with subtype C1, subtype C2 had a remarkably higher riskscore (Figure 3D). Further, we categorized patients into a high‐risk group and a low‐risk group in accordance with the median value as a cut‐off value. PCA analysis showed an obvious dimension between the low‐risk and high‐risk groups (Figure 3E). The distribution plot of the risk of NRG score revealed that the death rate was higher in high‐risk group (Figure 4A). Similarly, the Kaplan–Meier analysis showed that high‐risk score group had a poorer prognosis, compared with low‐risk score group (log‐rank test *p* < 0.001; Figure 4B). ROC curves further confirmed the predictive performance of this prognostic model. The area under the curves (AUCs) was 0.68, 0.64 and 0.58 in 1‐year, 3‐year and 5‐year OS rate, respectively (Figure 4C). We also validate the accuracy of this risk model in GSE37745 cohort (*n* = 196) and GSE3141 (*n* = 111). The patients were divided into low‐risk group and high‐risk group according to the formula used for the training set. The distribution plot of risk showed that risk score was associated with survival status (Figure 4D; Figure S2A). In addition, the Kaplan–Meier analysis showed a negative relationship between risk score and prognosis (log‐rank test *p* < 0.05; Figure 4E; Figure S2B). The time‐dependent ROC curves showed that the NRG score had relatively high AUC values (Figure 4F; Figure S2C), suggesting that the NRG score had excellent ability to predict the survival of lung cancer patients.

### 3.4 Establishment of a prognostic nomogram model

**FIGURE 5:**
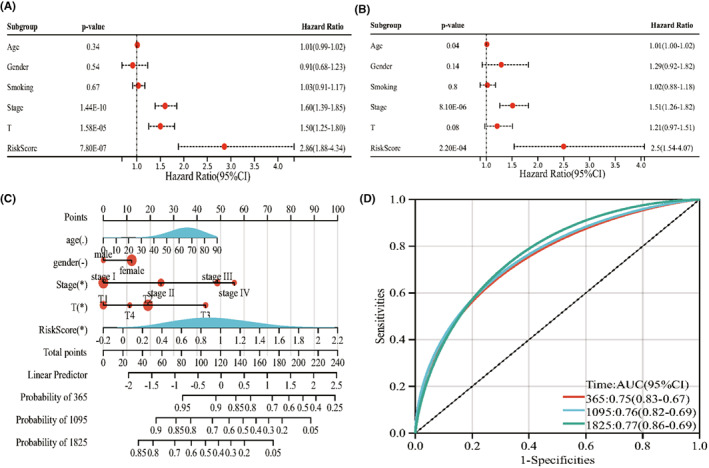
Correlation between risk score and prognosis. (A) Univariate Cox regression analysis of NRG score and clinicopathological features. (B) Multivariate Cox regression analysis of NRG score and clinicopathological features. (C) Establishment of nomogram model. (D) ROC curves of nomogram model at 1 year, 3 year, 5 year

Further, we performed univariate and multivariate Cox regression analyses to test whether the prognostic model could predict the prognosis independently. Univariate Cox regression analysis indicated that tumour stage (*p* < 0.001), tumour size (*p* < .001), and risk score (*p* < 0.001) were hazard factors (Figure 5A). Multivariate Cox regression analysis also confirmed that tumour stage (*p* < 0.001) and risk score (*p* < 0.001) were independent prognostic indicators (Figure 5B). Further, we integrated NRG score with clinicopathological features to establish a nomogram model that can more accurately and steadily assess the prognosis (Figure 5C). The AUCs for the 1‐year, 3‐year and 5‐year OS rate were 0.75, 0.76 and 0.77, respectively (Figure 5D).

### 3.5 Relationship between the NGR signature and tumour immune microenvironment

**FIGURE 6:**
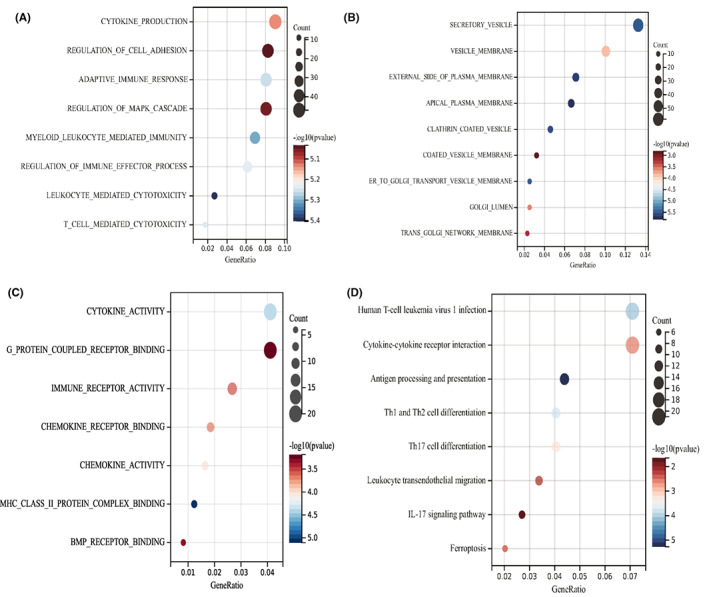
Functional enrichment analyses of low‐risk and high‐risk groups. (A) GO biological process analysis of low‐risk and high‐risk groups. (B) GO cellular component analysis of low‐risk and high‐risk groups. (C) GO molecular function analysis of low‐risk and high‐risk groups. (D) KEGG pathway analysis of low‐risk and high‐risk groups

**FIGURE 7:**
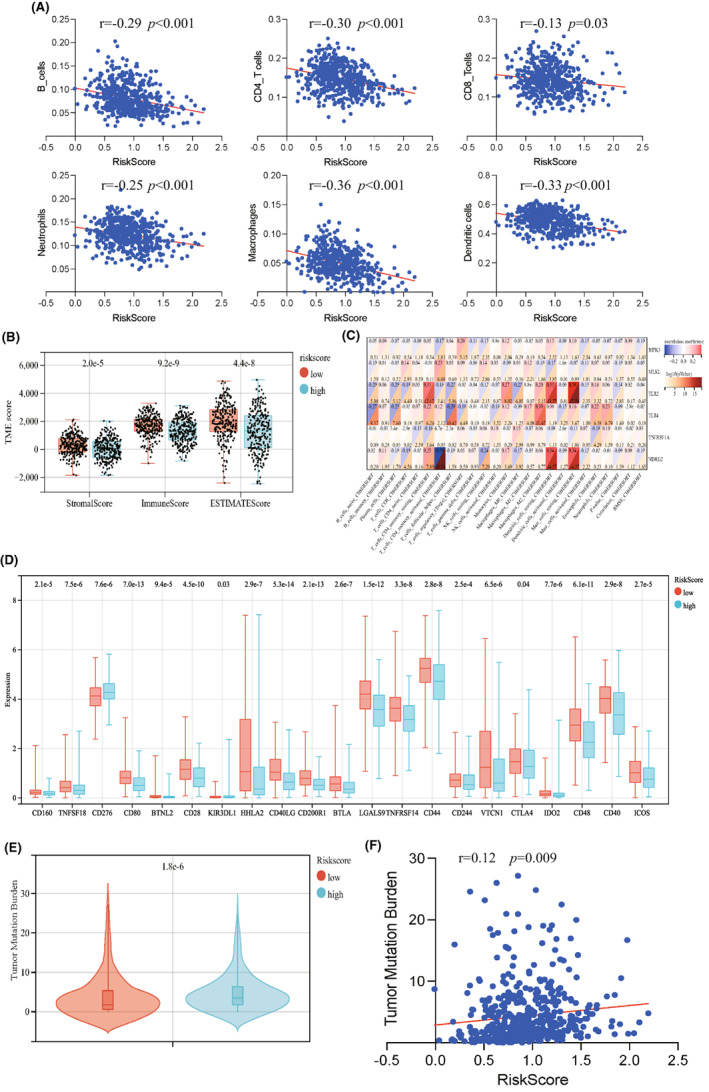
Relationship between NGR signature and tumor immune microenvironment. (A) Correlation between risk score and immune cells. *p < 0.05; ***p < 0.001. (B) Stromal scores, immune scores, and ESTIMATE score of low‐risk and high‐risk groups. ***p < 0.001. (C) Correlation between immune cells and six necroptosis‐related genes. (D) Expression levels of immune check points in the low‐risk and high‐risk groups. (E) Tumor mutation burden of low‐risk and high‐risk groups. ***p < 0.001. (F) Correlation between risk score and tumor mutation burden. *p < 0.05; **p < 0.01; ***p < 0.001

To explore the potential biological function of necroptosis pattern, we identified 635 DEGs between high‐risk group and low‐risk group (Figure S3) and performed functional enrichment analyses. GO biological process analysis revealed that these DEGs were mainly enriched in the regulation of immunity, including cytokine production, regulation of immune effector process, leukocyte mediated cytotoxicity and T‐cell mediated cytotoxicity (Figure 6A). Transcription proteins were mostly located in secretory vesicle, external side of plasma membrane and apical plasma membrane (Figure 6B). Cellular components molecular functions were involved in cytokine activity, immune receptor activity, chemokine receptor binding and chemokine activity (Figure 6C). KEGG analysis showed that DEGs may play an important role in cytokine‐cytokine receptor interaction, antigen processing and presentation, Th1 and Th2 cell differentiation, Th17 cell differentiation and leukocyte transendothelial migration (Figure 6D). Then, we investigated the correlation between the risk score and immune infiltration. TIMER algorithm revealed that risk score was negatively associated with immune cells, including B cells, CD4^+^ T cells, CD8^+^ T cells, neutrophils, macrophages and dendritic cells (Figure 7A). Moreover, stromal scores, immune score and ESTIMATE score were significantly increased in low‐risk group, compared with high‐risk group (Figure 7B). We also evaluate the correlation between the six necroptosis‐related genes and the abundance of immune cells. The result showed that multiple immune cells were associated with the six NRGs (Figure 7C). More importantly, the expression levels of 19 immune checkpoints were increased in low‐risk group, compared with high‐risk group (Figure 7D).

Gene mutation is an important factor in lung cancer initiation and progression.^26^ Therefore, we investigated whether there was a relationship between risk score and tumour mutation burden (TMB). The result showed that TMB was increased in high‐risk group, compared with low‐risk group (Figure 7E). In addition, spearman correlation analysis indicated that the NRG‐related risk score was positively related with the TMB (Figure 7F).

### 3.6 Comparison between risk model and other models

To evaluate our risk model, we compared the prognostic results obtained from a single NRG. The results showed that the expression of TLR2, TNFRSF1A and NDRG2 was associated with the prognosis of TCGA‐LUAD cohort (log‐rank test *p* < 0.05, Figure S4A). However, the AUCs of the ROC curves for 1‐year, 3‐year and 5‐year OS rate showed less predictive power than that of our risk model (Figure S4B). In addition, in GSE37745 cohort, the expression of TLR2, TNFRSF1A and NDRG2 was not correlated with prognosis (log‐rank test *p* > 0.05, Figure S4C). Taken together, these results suggest that our risk model has better accuracy and stability and can reflect the prognosis of NSCLC patients more comprehensively.

## 4 DISCUSSION

Necroptosis is a caspase‐independent cell death that shares features of apoptosis and necrosis.^27^ Similar to necrosis, the morphologic characteristics of necroptosis include swelling of organelles, condensation of chromatin, increased cell volume and disruption of the plasma membrane. More importantly, necroptosis is a regulated and controlled cell death manner.^28^ These indicate that necroptosis may become a promising approach to overcome apoptosis resistance in cancer therapy. Recently studies have reported that necroptosis‐associated genes, such as RIPK1,^29^ RIPK3,^30^ MLKL,^31^ HMGB1,^32^ may trigger strong immune responses in multiple cancers. However, those work only focused on the role of an NRG in one type tumour, the overall immune responses mediated by the synthetic effects of multiple NRGs in lung cancer are little known.

In this study, we identified two necroptosis‐related patterns on the basis of seventeen NRGs. Compared with subtype C1, subtype C2 showed a significantly worse prognosis. Subtype C2 tended to have a more advanced stage and a higher recurrence rate. Moreover, GVSA enrichment analysis showed that subtype C1 was more activated in immune‐associated pathways, including cytokine receptor interaction, chemokine signalling pathway, TGF‐β signalling pathway, antigen processing, Toll‐like and NOD‐like receptor signalling pathways, JAK–STAT signalling pathway, natural killer cell‐mediated cytotoxicity, T‐ and B‐cell receptor signalling pathway. Consistent with this result, the levels of TME infiltrations were much higher in subtype C1. These findings suggest that NRGs may be predictors of clinical outcome and immunotherapeutic response in lung cancer.

We also established an effective NRG prognostic signature and demonstrated its predictive ability in TCGA‐LUAD, GSE37745 and GSE3141 cohorts. The Kaplan–Meier curves showed that risk score was negatively associated with overall survival. Univariate and multivariate Cox regression analyses indicated that this signature may be used for prognosis stratification of NSCLC patients, which further confirmed by a quantitative nomogram. Necroptosis‐related prognostic model has been constructed in breast cancer,^33^ kidney renal clear cell carcinoma^34^ and stomach adenocarcinoma,^35^ but it has not been elucidated in lung cancer. RIPK3 and MLKL are crucial molecular components in necroptosis. The binding of RIPK3 and MLKL leads to the translocation of MLKL to the plasma membrane, therefore initiating the process of necroptosis.^27^ Inhibitors of RIPK3 and MLKL may protect cell from necroptosis induced by drugs.^36^, ^37^ Decreased expression of RIPK3 or MLKL was found to be associated with worse disease free survival of lung cancer.^12^ Moreover, activation of RIPK3/MLKL‐dependent necroptosis increased the sensitivity of gefitinib in NSCLC.^38^ TLR4 belongs to Toll‐like receptor family, which promote antigen presentation to trigger adaptive immune response. It may directly activate necroptosis through interaction with TRIF‐ RIPK3 complex.^39^ Upregulation of TLR4 has been found to enhance the immune response to chemotherapy in NSCLC.^40^

In addition, our study found that low‐risk group had a significantly higher levels of tumour‐infiltrating lymphocytes. Immune cells, including M1 macrophages, dendritic cells (DCs), natural killer (NK) cells and cytotoxic T cells, have an important function on innate and adaptive immune response.^27^ Necroptosis may release damage‐associated molecular patterns (DAMPs) into the tissue microenvironment,^41^ and stimulate phagocytic cells to produce pro‐inflammatory cytokines, eventually inducing robust adaptive immune responses.^42^, ^43^ RIPK3 has been reported to be critically required for the expression of inflammatory cytokines in DCs.^44^ Deficiency of RIPK3 impaired the antigen cross‐presentation ability of DCs to CD8^+^ T cells.^45^ Meanwhile, treatment with MLKL mRNA enhanced the antitumor capacity of CD4^+^ and CD8^+^ T cells.^46^ Our study also found that low‐risk group had much higher levels of 19 immune checkpoints. Immune checkpoint blockers have made great progress in oncology and established a new subfield of immuno‐oncology.^47^ Immunotherapy, especially against immune checkpoints, such as PD1 or cytotoxic T lymphocytes associated protein 4 (CTLA4), has been approved for the treatment of multiple solid malignancies, including lung cancer.^48^, ^49^, ^50^ Patients with high expression of PD1 or CTLA4 showed good response to anti‐PD‐1 or anti‐CTLA4 immune checkpoint inhibitors.^50^ Thus, we concluded that subtype C1 and low‐risk group may benefit from immunotherapy.

Our work still had several limitations. This study was retrospective, and all the data were from the public database. Large‐scale, multicentre and prospective work are needed to confirm these results. Additional cytological experiments are deserved to perform to verify our findings.

## 5 CONCLUSION

Our study revealed that the NRG model may be used to evaluate the prognosis, TME and TMB of NSCLC patients. We also found that NRG score is an independent prognostic factor for NSCLC patients and may be used to predict the efficacy of immunotherapy for lung cancer.

## AUTHOR CONTRIBUTIONS

**Ju‐ji Dai:** Formal analysis (equal); investigation (equal); project administration (equal). **Yangyang Fu:** Formal analysis (equal); funding acquisition (lead); investigation (equal); project administration (equal); supervision (lead); writing – original draft (lead).

## CONFLICT OF INTEREST

The authors declare that there is no conflict of interest.

## Supporting information


AppendixS1

AppendixS2

## Data Availability

The datasets analyzed for this research can be found in the TCGA‐LUAD project (https://portal.gdc.cancer.gov/), GEO database (GSE37745) (https://www.ncbi.nlm.nih.gov/geo/query/acc.cgi?acc=GSE37745) and GEO database (GSE3141) (https://www.ncbi.nlm.nih.gov/geo/query/acc.cgi?acc=GSE3141).
