# Supplementary material for: Identification of necroptosis‐related gene signature and characterization of tumour microenvironment infiltration in non‐small‐cell lung cancer
Source: J Cell Mol Med. 2022 Jul 24;26(17):4698–709. doi: 10.1111/jcmm.17494 (PMC9443942; doi:10.1111/jcmm.17494)
Supplement: Supplementary file 1 — AppendixS1 [file JCMM-26-4698-s002.docx]

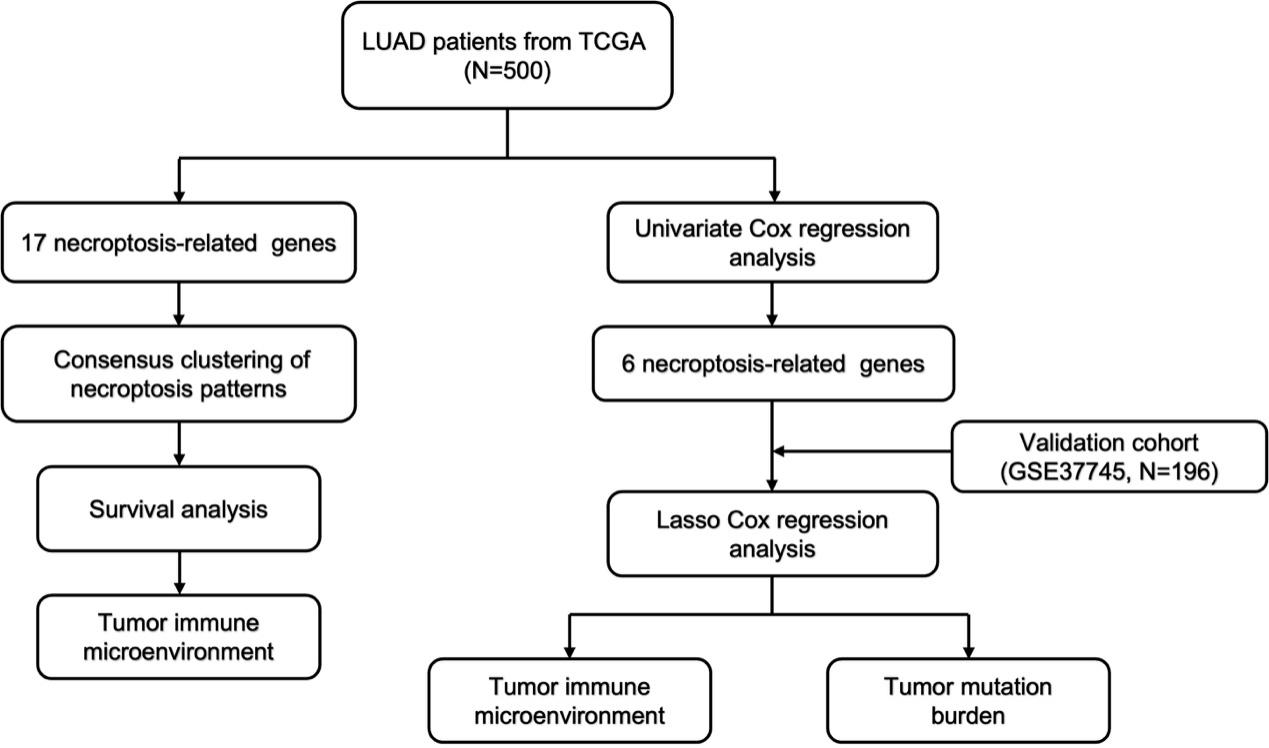


Supplementary figure 1 Flow chart of the study.


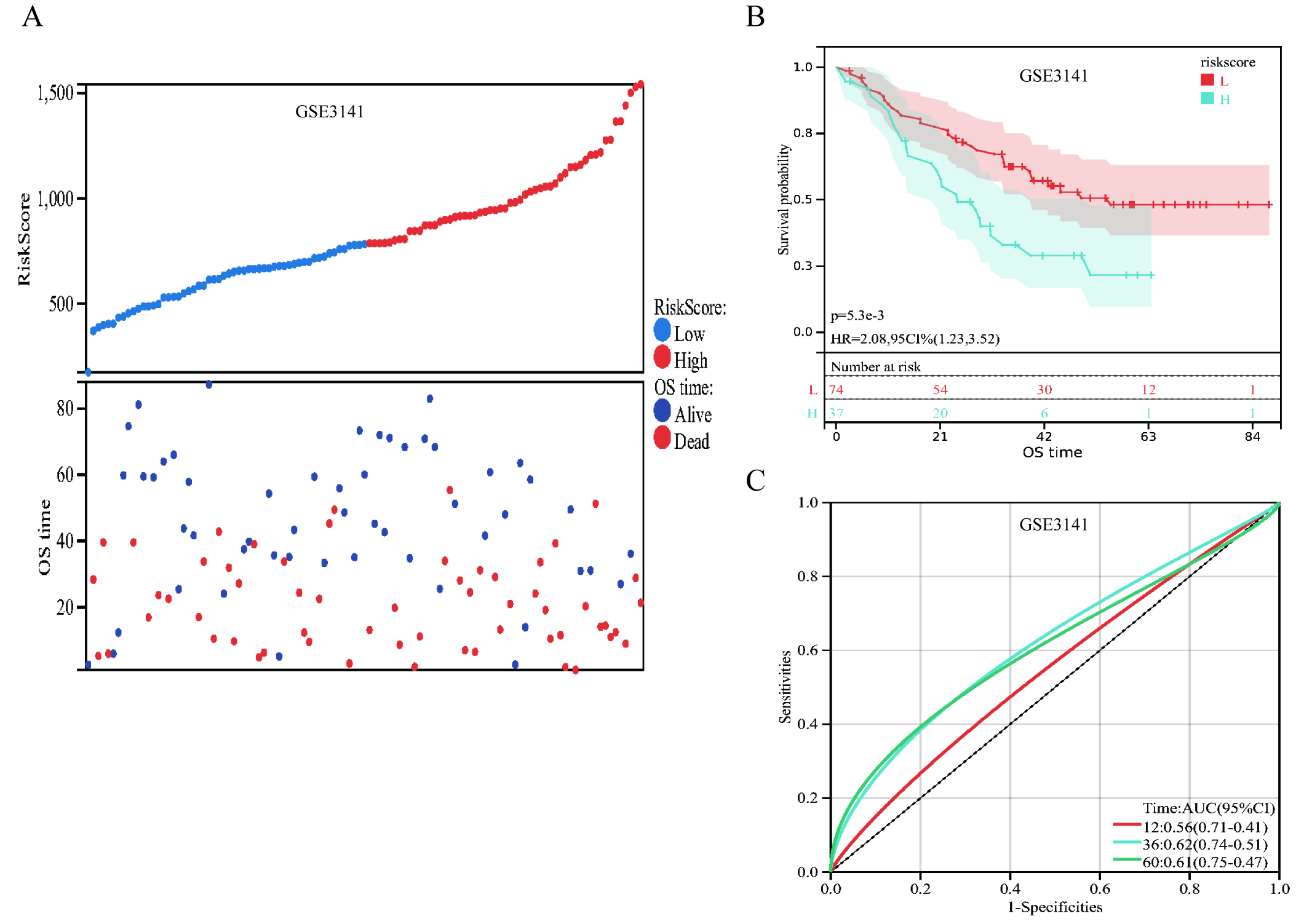


Supplementary figure 2 Validation of NRG score in testing set GSE3141. (A) Distribution of risk score and survival status in GSE3141 set. (B) Kaplan-Meier curves of low-risk and high-risk groups in GSE3141 set. Logrank *p*<0.05. (C) ROC curves of NRG score in GSE3141 set. ^*^*p*<0.05; ^**^*p*<0.01; ^***^*p*<0.001.


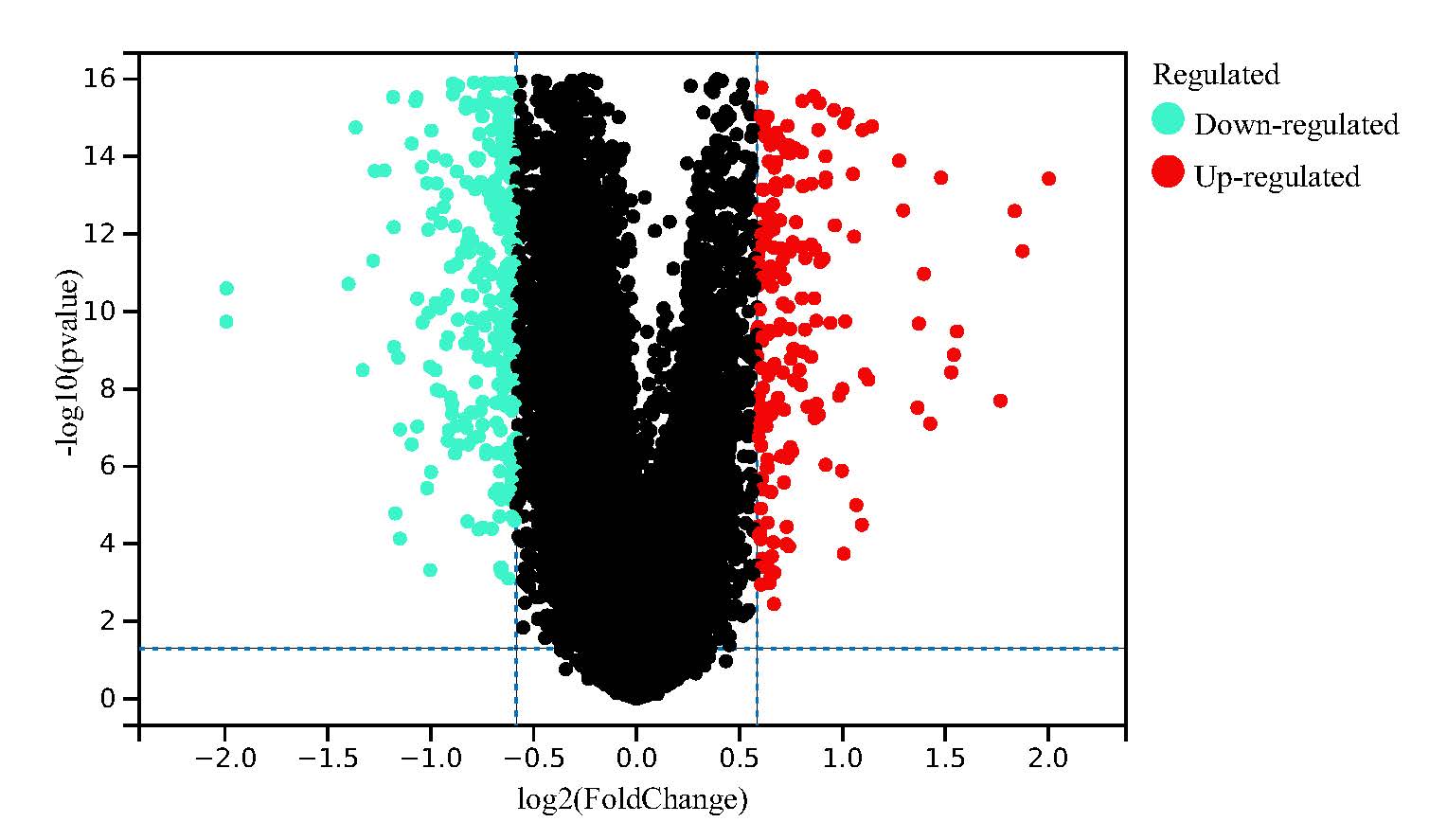


Supplementary figure 3 Differentially expressed genes of low-risk and high-risk groups.


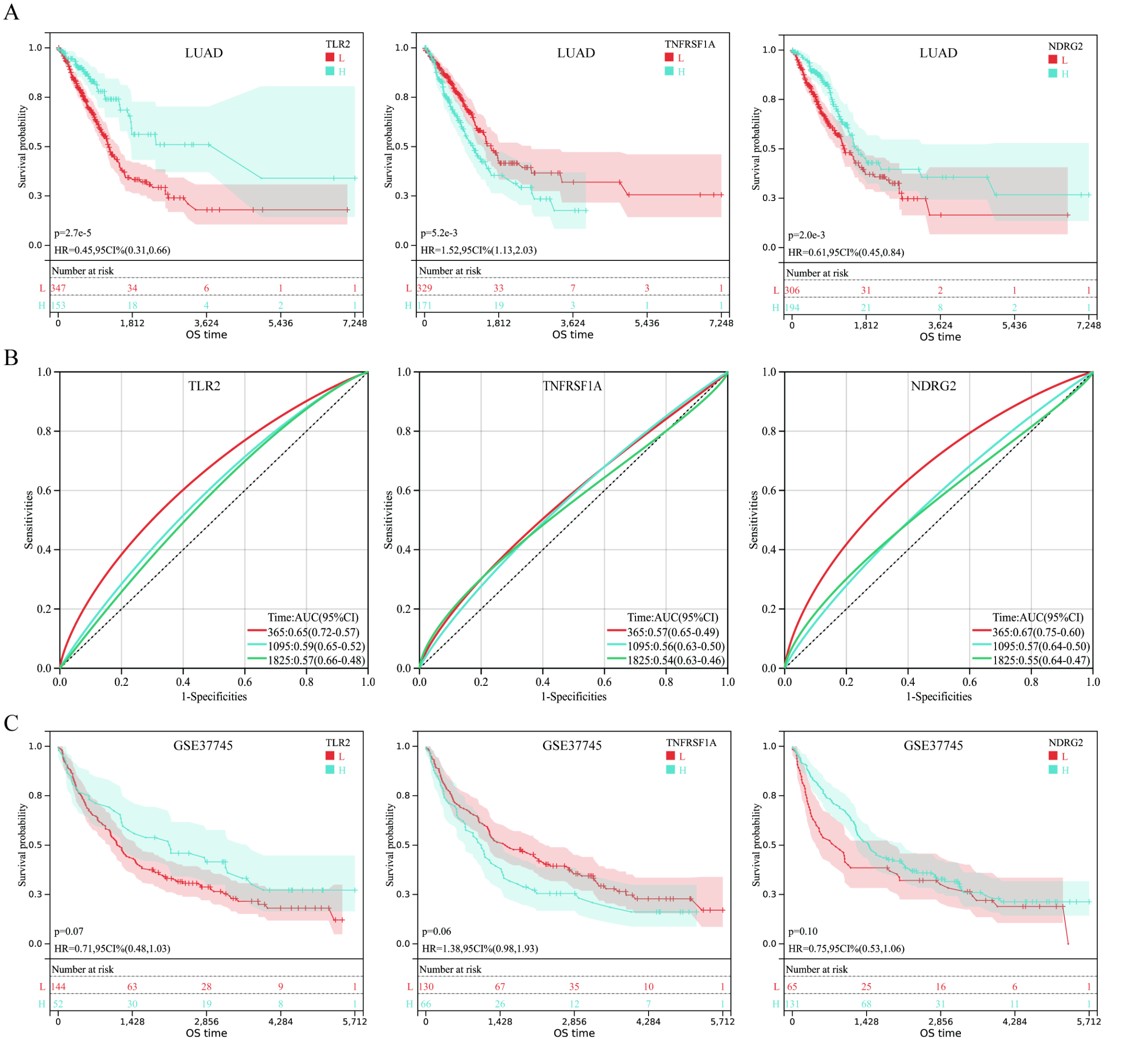


Supplementary figure 4 Kaplan-Meier and ROC curves of TLR2, TNFRSF1A, and NDRG2 in trainning and testing set. (A) Kaplan-Meier curves of TLR2, TNFRSF1A, and NDRG2 in TCGA-LUAD cohort. Logrank *p*<0.05. (B)ROC curves of TLR2, TNFRSF1A, and NDRG2 in TCGA-LUAD cohort. (C) Kaplan-Meier curves of TLR2, TNFRSF1A, and NDRG2 in GSE37745 cohort.
